# Graph-Based Visual Manipulation Relationship Reasoning Network for Robotic Grasping

**DOI:** 10.3389/fnbot.2021.719731

**Published:** 2021-08-13

**Authors:** Guoyu Zuo, Jiayuan Tong, Hongxing Liu, Wenbai Chen, Jianfeng Li

**Affiliations:** ^1^Faculty of Information Technology, Beijing University of Technology, Beijing, China; ^2^Beijing Key Laboratory of Computing Intelligence and Intelligent Systems, Beijing, China; ^3^School of Automation, Beijing Information Science and Technology University, Beijing, China; ^4^Faculty of Materials and Manufacturing, Beijing University of Technology, Beijing, China

**Keywords:** relationship reasoning, graph convolution network, grasping order, robotic manipulation, object-stacking scene

## Abstract

To grasp the target object stably and orderly in the object-stacking scenes, it is important for the robot to reason the relationships between objects and obtain intelligent manipulation order for more advanced interaction between the robot and the environment. This paper proposes a novel graph-based visual manipulation relationship reasoning network (GVMRN) that directly outputs object relationships and manipulation order. The GVMRN model first extracts features and detects objects from RGB images, and then adopts graph convolutional network (GCN) to collect contextual information between objects. To improve the efficiency of relation reasoning, a relationship filtering network is built to reduce object pairs before reasoning. The experiments on the Visual Manipulation Relationship Dataset (VMRD) show that our model significantly outperforms previous methods on reasoning object relationships in object-stacking scenes. The GVMRN model is also tested on the images we collected and applied on the robot grasping platform. The results demonstrated the generalization and applicability of our method in real environment.

## 1. Introduction

Grasping is one of the most important means for the robot to interact with the environment. As the grasping scenes become more and more complex, for example, from a single object simple scene to a multi object-stacking scene, the demand for intelligent grasping is also increasing. That is to say, when interacting with the environment, the robot can gradually obtain the ability to perceive and understand the surrounding scenes, guide its decision-making and actions, and achieve advanced intelligence. Traditionally, robotic manipulation focuses on robotic grasping detection (Lenz et al., 2015; Redmon and Angelova, 2015) and grasping pose estimation (ten Pas et al., 2017; Ni et al., 2019), ignoring the interaction and influence between objects. However, it is necessary and important for the robot to obtain the relationships between objects and then to realize the interaction with the environment at a higher level (Yang et al., 2018; Wu et al., 2019; Zhang et al., 2020).

**Figure 1:**
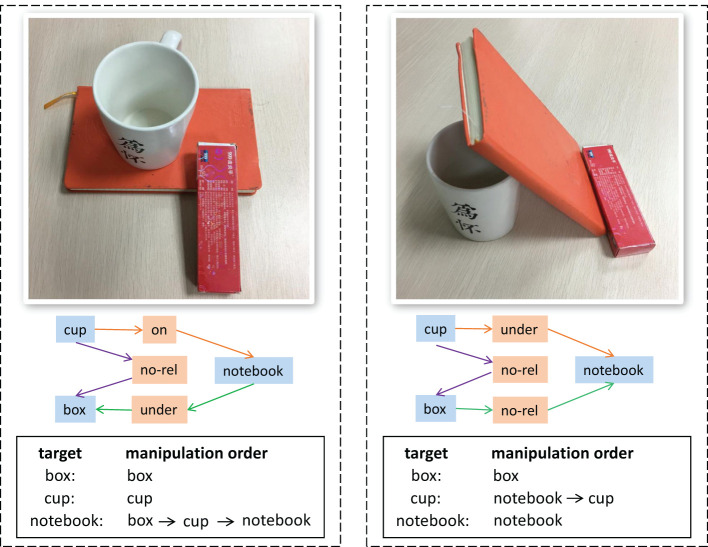
The influence of different relationships between objects on manipulation order. The first row is the object-stacking scene, the second is the object relationship graph, and the third is the manipulation order of target objects.

As shown in Figure 1, the relationships between objects have a great influence on the manipulation order. Even though both two images contain the same objects (a cup, a box and a notebook), it is the relationships between objects that determine how to grasp the target object correctly and effectively. Taking grabbing the notebook as an example. In the right part of the figure, the notebook can be grabbed directly. However, in the left part, the cup and box need to be moved away first. If ignoring the relationships between objects in the scene, we may break the cup when grabbing the notebook directly. Therefore, it is crucial to get the object relationships to reason the manipulation order.

Lu et al. (2016) proposed the concept of visual relationship (VR). He pointed out that visual relationship is a triple consisting of object 1, object 2 and a predicate, and he proposed a visual relation detection model using language priors. He also made a large-scale visual relation detection (VRD) dataset for visual relationship reasoning. The limitations of this method are independent prediction of each relationship and lack of context information. Xu et al. (2017) proposed a graph-based model to transmit context information in the form of iterative message passing. Yang et al. (2018) proposed an attentional graph convolutional model that obtains the proposal relationships according to the relevance of object category, and integrates context information by graph convolution network. Yao et al. (2018) used graph convolution network and LSTM to generate visual relationships for image captioning.

Rosman and Ramamoorthy (2011) proposed the concept of spatial relationship (SR). He constructed the contact point network of the topological structure based on point cloud to describe spatial relationship by locating the contact points between objects. Three kinds of support relations between objects are considered to learn spatial relations in the cluttered environment (Panda et al., 2016), so as to help the robot get manipulation order. The spatial relationship is applied on manipulation actions to improve the classification accuracy of actions (Ziaeetabar et al., 2017).

The relation of guiding the robot to grab orderly is defined as visual manipulation relationship (VMR) (Zhang et al., 2020), which aims to solve the problem that the robot can grasp the target object correctly and effectively in the object-stacking scenes. Zhang et al. (2020) built a visual manipulation relationship reasoning network based on RGB images to construct the manipulation relationship tree, and established a visual manipulation relationship dataset (VMRD). VMR is different from VR and SR in that the later two methods cannot directly guide the robot to grasp. Park et al. (2020) combined object detection, grasping detection and object relation reasoning to achieve a high grasp success rate in cluttered scenes. Wu et al. (2019) used the point cloud information and RGB information to obtain manipulation order. Han et al. (2020) proposed an optimal sorting position and pose estimation network to sort disorderly stacked parcels in logistics environment.

Although some recent works have been done to solve the problem of visual manipulation relationship reasoning, it is still a challenge to extract the relationships between objects effectively and accurately on images. Visual manipulation relationship reasoning networks (VMRN) (Zhang et al., 2020) and Multi-task CNN (Zhang et al., 2019) use convolution layers and fully connected layers for reasoning. If they are applied to the relationship reasoning of all objects in the image, it is inevitable to traverse each pair of relationships. Traversing all objects pairs is an effective method to train the relation reasoning model when the number of objects is small. As the number of objects increases, redundant information is also increasing greatly. To be brief, convolutional neural networks (CNNs) work weakly for some non-Euclidean structures such as relationship networks. Kipf and Welling (2016) proposed graph convolution network (GCN) to extract and learn features from non-Euclidean data, and solved semi-supervised classification for node and edge structures. Recently, graph convolution networks (GCNs) have been used to solve the problem of relational reasoning (Xu et al., 2017; Yao et al., 2018). Therefore, different from VMRN and Multi-task CNN, our model focuses on representing the information in the image as the objects and their relationships, which can directly reason object relationships and manipulation order by graph convolution network. We also build a relation filtering network to prune the object pairs that are unlikely to have relationships to improve the efficiency of relational reasoning.

Our contributions are summarized below:

- A novel end-to-end graph-based visual manipulation relationship reasoning network is proposed to directly reason the object relationships and the manipulation order based on RGB image.
- A relation filtering network is built to improve the efficiency and accuracy of relationship reasoning by filtering out the non-contact object pairs before relationship reasoning.
- GCN is used to realize the synchronization prediction of all the object relationships in the image.

## 2. Method

**Figure 2:**
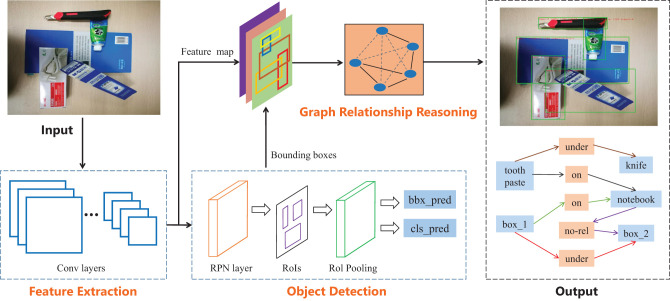
Overview of our end-to-end object relationship reasoning architecture. The model mainly includes three modules: feature extraction, object detection and graph relationship reasoning. The feature extraction module consists of a stack of convolution layers (VGG or ResNet) that output feature maps. The object detection module is used to generate the bounding boxes of all objects. The feature maps and the bounding boxes are used to predict the object relationships and the manipulation order through the graph relationship reasoning module.

The goal of our method is to learn the visual relation reasoning model from RGB images, and directly output object relationships in the object-stacking scenes and manipulation order of grasping the target object. Figure 2 shows the architecture of our method, which first extracts features and detects objects from RGB images, and then transfers the feature maps and the bounding boxes of objects to the relationship reasoning module for training. To directly output the manipulation order according to the relationships between objects, we define the following three types of visual manipulation relationships: object A is on object B (*on*), object A is under object B (*under*), and object A and object B have no relationship (*no*_*rel*). In this method, we chose VGG16 and Resnet101 network as the backbone network to extract features. To obtain good detection accuracy, Faster R-CNN (Ren et al., 2015) is used to extract the object proposals in the object detection module, for there are small objects to be detected in the task.

**Figure 3:**
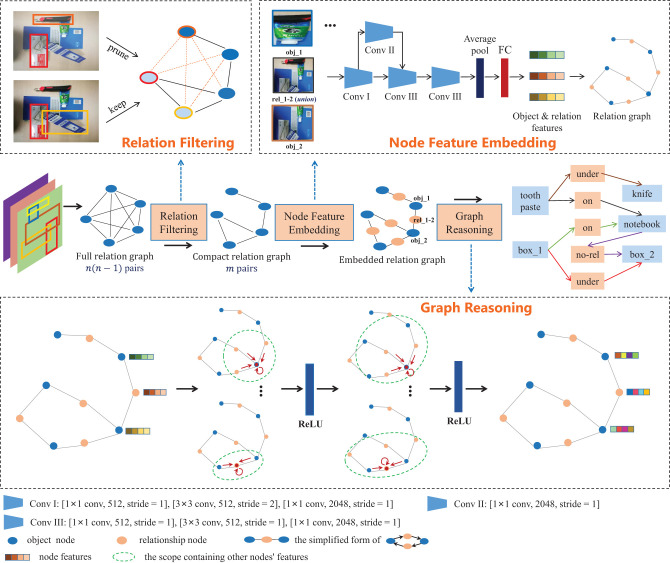
Graph-based relationship reasoning module. It includes three parts: relation filtering, node feature embedding and graph reasoning. Relation filtering reduces the number of object pairs with possible relationships from *n*(*n*−1) to *m*. Node feature embedding is used to generate node features and form the embedded relation graph. Graph reasoning is used to update node features and predict object relationships.

Figure 3 shows the work flowchart of graph-based relationship reasoning, which is the core module of our method, including three parts: relation filtering, node feature embedding and graph reasoning. To improve the efficiency of relational reasoning, unrelated object pairs are filtered out first. GCN is used to update the node features and infer the relationships between objects.

### 2.1. Relation Filtering

Relation filtering network is used to prune unrelated object pairs from the full relation graph and generate the compact one. Among *n* objects being detected, there will be *n*(*n*−1) possible relationships. Actually, many object pairs do not have relations. As the number of objects increases, the number of unrelated object pairs increases rapidly. So, we build a relation filtering network to solve this problem. Before the coordinates of *n* boxes are transferred into the relation reasoning module, some unrelated object pairs are deleted in advance according to the position information of object proposals. We traverse all object pairs and judge whether two proposal boxes (*o*_*i*_, *o*_*j*_) are filtered according to the following equations:

(1) $$L(o_{i},o_{j})=\{\begin{array}{ll} 0 & \text{if }{\text{S}}_{\text{inter}}({\text{o}}_{\text{i}},{\text{o}}_{\text{j}})>\text{0}\\ \frac{h(o_{i},o_{j})}{{\text{C}}_{\text{img}}} & \text{if }{\text{S}}_{\text{inter}}({\text{o}}_{\text{i}},{\text{o}}_{\text{j}})\text{=0} \end{array}\;i\neq j$$

(2) $$F(o_{i},o_{j})=\{\begin{array}{ll} 0 & \text{if L}({\text{o}}_{\text{i}},{\text{o}}_{\text{j}})>\delta \\ 1 & \text{if L}({\text{o}}_{\text{i}},{\text{o}}_{\text{j}})\leq \delta \end{array}\;i\neq j$$

where *S*_*inter*_ is the function that computes the intersection area between two boxes, *h*(*o*_*i*_, *o*_*j*_) represents the shortest distance between two non-intersect bounding boxes (rectangular boxes), and C_img_ represents the length of the image and it is a constant. *L*(*o*_*i*_, *o*_*j*_) is defined to normalize *h*(*o*_*i*_, *o*_*j*_) and its value is between 0 and 1. We define *F*(*o*_*i*_, *o*_*j*_) as a filter function to determine whether the object pair is filtered out. We set *F* = 1 and the object pair is retained if *L*(*o*_*i*_, *o*_*j*_) is less than or equal to the threshold δ, otherwise *F* = 0 and the object pair is filtered out. This soft filtering is realized by setting different values to δ. In the experiment, we set δ = 0.05. Object pairs with possible relationships is updated as follows:

(3) $$\begin{array}{r} q\left(o_{i},o_{j}\right)=F\left(o_{i},o_{j}\right)\cdot p\left(o_{i},o_{j}\right) \end{array}$$

where *F*(*o*_*i*_, *o*_*j*_)∈{0, 1}, *p* is the original object pairs set with *n*(*n*−1) dimensions, and *q*(*o*_*i*_, *o*_*j*_) is the object pair set with *m* dimensions. As shown in “Relation Filtering" of Figure 3, the orange dotted line indicates that the relationship of the object pair is filtered out, while the black solid line indicates that the relationship is retained.

### 2.2. Node Feature Embedding

As we see in the above subsection, the full relation graph is transformed into the compact relation graph through relation filtering. The node feature embedding module generates node features, including object features and relation features, and embeds both of them into the compact relation graph to form the embedded graph for reasoning. In this process, relation nodes are inserted between every two connected object nodes in the compact graph. Considering the noncommutativity of the relationship, we use *Rel*_*j*−*i*_ and *Rel*_*i*−*j*_ to represent two directed relationships of *Obj*_*i*_ to *Obj*_*j*_ and *Obj*_*j*_ to *Obj*_*i*_, respectively. We insert two directed relation nodes between each object pair with possible relationship. Here, the relation node is adopted to represent the relationship of objects instead of using edge features, because our relation features are multi-dimensional and rich, and the relation features can be updated according to the node feature updating mechanism in graph reasoning.

When generating the object and relation features in node feature embedding, we first define the union box as the smallest rectangle that can cover two smaller bounding boxes. The union box of two objects (*o*_*i*_, *o*_*j*_) is used to calculate relation node features, and the bounding box of the object is used to calculate object node features. According to the bounding boxes and union box of two objects, the initial features are cropped from feature maps with the size of 38 × 50 and transformed into the pooling features with the same size of *H*× *M* (7 × 7). A series of convolution operations are conducted on the pooling features, and average pooling is conducted. The final object and relation features are generated through a fully connected layer. They are embedded into the relation graph to form relation nodes and object nodes with their different features. In the test, the embedded relation node with higher confidence is selected as the predicted relationship of the object pair.

### 2.3. Graph Reasoning

The visual relationship reasoning of objects is conducted on the graph *G* = (*A, X*), which consists of a sparse structure *A* and the node feature vector *X*. Two GCN layers are built to update node features and predict object relationships, as shown in the graph reasoning part of Figure 3. In the graph training, there are two types of nodes: the object nodes and the relationship nodes that are inserted between object nodes. The node feature update for this embedded structure can be expressed as follows:

(4) $$\begin{array}{r} x_{i}^{\left(l+1\right)}=\delta \left(x_{i}^{l}+\underset{j\in N_{i}}{\sum }D^{-\frac{1}{2}}AD^{\frac{1}{2}}x_{j}^{l}W_{obj}^{l}\right)\;x_{i}\in x_{obj} \end{array}$$

(5) $$\begin{array}{r} x_{i}^{\left(l+1\right)}=\delta \left(x_{i}^{l}+\underset{j\in N_{i}}{\sum }D^{-\frac{1}{2}}AD^{\frac{1}{2}}x_{j}^{l}W_{rel}^{l}\right)\;x_{i}\in x_{rel} \end{array}$$

respectively for the object nodes (*x*_*obj*_) and for the relationship nodes (*x*_*rel*_). $W_{obj}^{l}$ and $W_{rel}^{l}$ are weight matrixs as well as updated parameters. $x_{i}^{l}$ is the node feature in *l*th layer, and *D* is the degree matrix that represents the number of connections between the current node and the other nodes. The updating features of each node are aggregated by its own node features and neighbor node features. There are three types of predicted relationship nodes (*on, under, no*_*rel*), therefore we use multi classification cross entropy function as the loss function of our network:

(6) $$\begin{array}{r} loss=-\overset{i}{\underset{n}{\sum }}y_{i}\log\left(\hat{y_{i}}\right) \end{array}$$

where $\hat{y_{i}}$ is the predicted result of the relationship node and *y*_*i*_ is the ground truth.

## 3. Experiments

### 3.1. Experimental Data and Evaluation Metrics

We evaluate our model on the Visual Manipulation Relationship Dataset (VMRD) (Zhang et al., 2020). In experiment, 90% of the 5, 185 images are used for training and the remaining 10% for testing, and the following four metrics:

**mAP** (mean Average Precision): This is a key performance metric in many multi-class object detection tasks, which is used to measure the detected results of all categories of objects.

**OR** (Object-based Recall): This metric is used to evaluate the recall on object pairs. The predicted result of the triplet (*Obj*_*i*_, *Rel*_*i*−*j*_, *Obj*_*j*_) is considered correct if the category of objects (*Obj*_*i*_, *Obj*_*j*_) and the manipulation relationship (*Rel*_*i*−*j*_) are both predicted correctly. We compute the average recall of three kinds of manipulation relationships (*on, under, no*_*rel*).

**OP** (Object-based Precision): Different from **OR** mentioned above, **OP** is the average precision of three kinds of manipulation relationships (*on, under, no*_*rel*). This metric is also valuated on object pairs.

**IA** (Image-based Accuracy): This metric is used to evaluate the accuracy based on the image. The prediction of the image is considered correct only if all triplets (*Obj*_*i*_, *Rel*_*i*−*j*_, *Obj*_*j*_) of the image are right. We also calculate accuracy of manipulation relationships when the number of the stacked objects is different.

### 3.2. Implementation Details

The two-stage training method is used for relation reasoning in our experiment. We first train the feature extraction and the object detection modules, and then fix the trained parameters to train graph-based visual manipulation relationship reasoning network. The initial learning rate is 0.001 for the first training stage. After 5 epochs, the learning rate decays to 0.0001. For the second training stage, the initial learning rate is 0.01.

We use Faster R-CNN to train the object detection module, and train the feature extraction module by using VGG16 network and Resnet101 network, respectively as backbone network based on the PyTorch re-implementation. In detail, the number of proposals from RPN is 256. For each proposal, we perform ROI Align pooling to get a 7 × 7 feature map, subsequently putting it into four convolution layers, one average pooling layer and one fully connected layer. Then, we can obtain the feature representation with 2048 dimensions for *n* objects and *m* relationships. When training the graph convolution network, two GCN layers are used to update the features of object nodes and relationship nodes.

To test performances different backbone networks, we trained two models (GVMRN), respectively on ResNet101 and VGG16. To evaluate the role of the relation filtering network, we also trained the model (GVMRN-RF) with it and the model (GVMRN) without it.

### 3.3. Results of Visual Manipulation Relationship Reasoning

**Figure 4:**
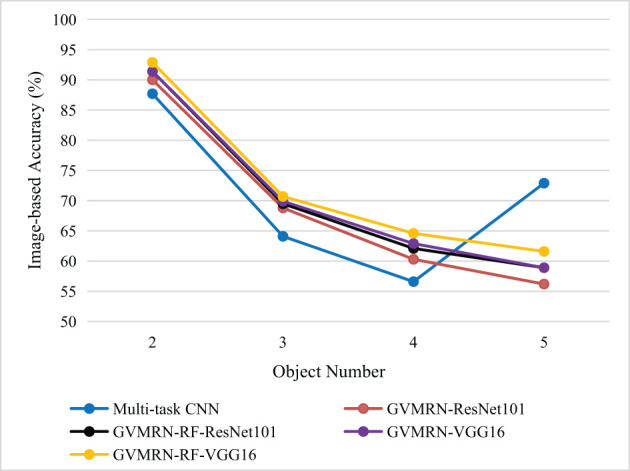
Image-based accuracy (IA) for different number of stacked objects.

**Figure 5:**
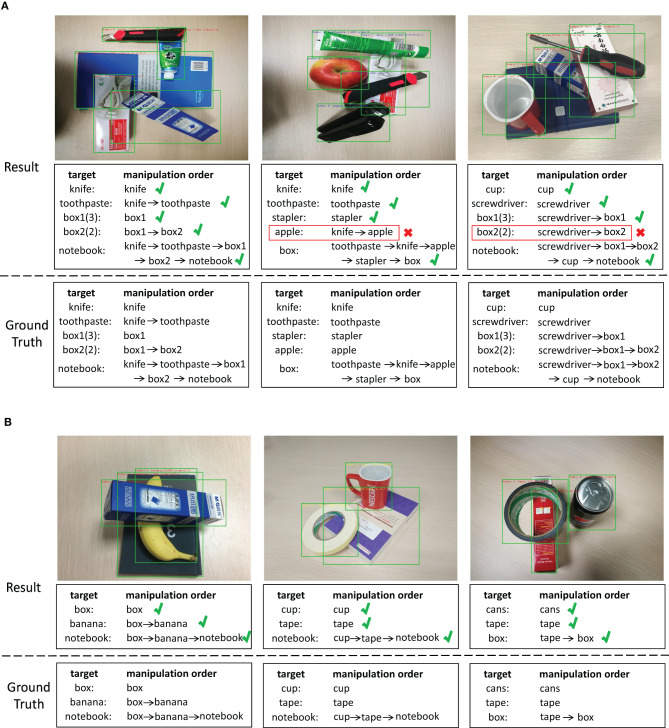
Examples of detection results of graph-based visual manipulation relationship reasoning network on the VMRD dataset. **(A)** Five stacked objects in one scene, and **(B)** three stacked objects in one scene. The first row shows detection results of the stacked objects, and each image contains the bounding boxes of the object, the confidence score and the manipulation order. The second row shows the manipulation order in the language form of words. The third row is the ground truth.

In experiment, we compared our network models with Multi-task CNN (Zhang et al., 2019) and VMRN (Zhang et al., 2020) on the VMRD dataset, and 525 images are used for testing. Tables 1, 2 show the experimental results from different aspects, and Figure 4 shows the comparison of different models by line chart. Some illustration reasoning results are given in Figure 5. We quantify its performance, respectively with mAP, OR, OP, and IA.

**Table 1:** Results of visual manipulation relationship reasoning.

**Feature network**	**Model**	**mAP**	**OR**	**OP**	**IA**	**Time (ms)**
ResNet101	Multi-task CNN (Zhang et al., 2019)	-	86.0	**88.8**	67.1	-
	VMRN (Zhang et al., 2020)	**95.4**	85.4	85.5	65.8	98
	GVMRN(ours)	94.5	86.3	87.1	68.0	102
	GVMRN-RF(ours)	94.6	**87.4**	87.9	**69.3**	**67**
	VMRN (Zhang et al., 2020)	94.2	86.3	88.8	68.4	71
VGG16	GVMRN(ours)	**95.4**	87.3	89.6	69.7	92
	GVMRN-RF(ours)	**95.4**	**89.1**	**89.7**	**70.9**	**58**

*Each bold value represents the optimal result for each metric for different methods*.

**Table 2:** Reasoning results of different numbers of objects.

**Feature network**		**Image-based accuracy (IA)**				
	**Model**	**Total**	**Object number per image**			
		**(%)**	**2**	**3**	**4**	**5**
ResNet101	Multi-task CNN (Zhang et al., 2019)	67.1	87.7	64.1	56.6	**72.9**
	VMRN (Zhang et al., 2020)	65.8	-	-	-	-
	GVMRN(ours)	68.0	90.0	68.8	60.3	56.2
	GVMRN-RF(ours)	**69.3**	**91.4**	**69.5**	**62.1**	58.9
	VMRN (Zhang et al., 2020)	68.4	-	-	-	-
VGG16	GVMRN(ours)	69.7	91.4	69.9	62.9	58.9
	GVMRN-RF(ours)	**70.9**	**92.9**	**70.7**	**64.6**	**61.6**

*Each bold value represents the optimal result for each metric for different methods*.

Table 1 shows the results of manipulation relationship reasoning of different models, and in general, our method has better performance. The performance of VGG16 is better than Resnet101, because VGG16 has more parameters and learns more information. For the mAP metric, almost all of the methods are around 95%, and VGG16 is slightly better than Resnet101. For the IA metric, the results in the 5th row and 8th row in Table 1 show that our models with relation filtering network have the better results.

For the Resnet101 part, the object-based recall (OR) and the image-based accuracy (IA) are higher than Multi-task CNN and VMRN, but Multi-task CNN has better performance in the object-based precision (OP). However, for the VGG16 part, our model (GVMRN) has better results for all the evaluation metrics. From above, We can conclude that the graph-based visual manipulation relationship reasoning network can better capture the information between objects in the image and infer manipulation relationships more effectively.

From the comparative results of GVMRN and GVMRN-RF in Table 1, we can see that the accuracy of manipulation relationship reasoning can be improved by using the relation filtering network. For ResNet101, the IA metric is improved from 68 to 69.3%, and for VGG16, the IA metric is improved by 1.2%. The OR metrics for the two backbone networks are both improved. However, there is no obvious change in the OP metric. The reason is that the relation filtering network deletes some object pairs (*no*_*rel*) before reasoning, for it is difficult to predict the relation (*no*_*rel*) to other relations (*on, under*), which helps to improve the recall of the no-relation category and further improve the OR metric.

As shown in the last column of Table 1, the speed of the models with VGG16 is generally faster than with Resnet101. Our models (GVMRN) with different backbone networks both have the longest time of relationship inference. For GVMRN-RF with ResNet101, due to relation filtering, the time of relationship inference is reduced from 102 to 67*ms* while for VGG16, the average time of predicting all relationships of one image is also reduced by 34*ms*. The experimental results strongly proves that the relational filtering mechanism can greatly reduce the time of relational reasoning.

Table 2 shows the results of manipulation relationship reasoning for different number of stacked objects. When there are only two objects in the image, the accuracy of manipulation relationship reasoning can reach about 90%. The GVMRN-RF model with VGG16 has the highest prediction accuracy of 92.9%. When the number of objects is five, the prediction accuracy drops to 61.6%. In contrast, the accuracy of Multi-task CNN is 72.9%. To better show the advantages of our model, we made a line chart of image-based accuracy, as shown in Figure 4. It can be found that the overall image-based accuracy decreases with the increase in the number of objects for all the models except Multi-task CNN. In other words, the more objects the image has, the more difficult to identify all the manipulation relationships correctly. Our models with GVMRN have better performance when the number of objects is less than five. Although our models do not show optimal results when the number of objects is five, it is worth noting that as the number of objects increases, the accuracy of our models doesn't drop sharply. Instead, it decreases more and more slowly, remaining relatively better results. Therefore, our graph-based visual manipulation relationship reasoning model can better learn the relation features and reason the manipulation relations between objects.

Figure 5 shows detection examples with the graph-based visual manipulation relationship reasoning network on the VMRD dataset. The results of five stacked objects and three stacked objects are shown, respectively in Figures 5A,B. As the first row shows, the network can directly output the category of the object, the bounding box, the confidence score and the manipulation order at the same time. In the second row, we list the prediction results of manipulation order, and the third row is the ground truth. For all the three examples of Figure 5A, the manipulation orders of the first example are predicted correctly. In the second example, the knife is wrongly inferred above the apple. So, the result shows that we need to grab the knife first, not the apple, which is inconsistent with the ground truth. A mistakenly recognized relationship is also generated in the third example. In Figure 5B, the manipulation orders are all correctly predicted. We can see that the results of Figure 5 verify the conclusions from the results of Table 2, that is, the fewer the stacked objects are, and the more likely the manipulation relationships are predicted correctly.

### 3.4. Performance Analysis on GVMRN

**Figure 6:**
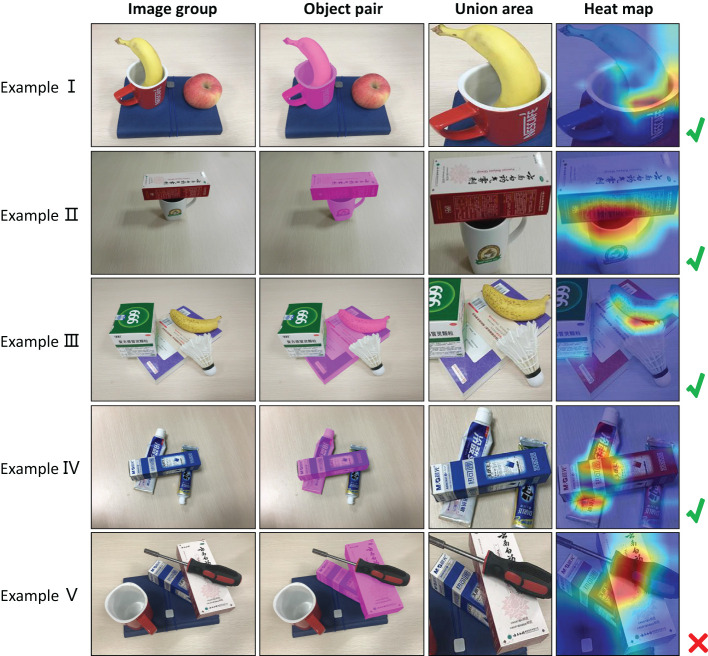
Feature visualization for graph-based visual manipulation relationship reasoning.

To further explain the effectiveness of our method, we use Gradient-weighted Class Activation Mapping (Grad-CAM) (Selvaraju et al., 2017) to visualize the feature maps. For the models that have been trained, Grad-CAM can indicate which part of the original image contributes to the classification result through a certain kind of gradient, and distinguish the regions of interest by using heat map. In the visualization of feature maps, we randomly select a pair of relationships in the image. Figure 6 shows feature visualization examples for graph-based visual manipulation relationship reasoning. The first column are the original images. The second column are the selected object pairs. The third column shows where to crop the union boxes of the selected object pairs. The ratio of the width and height of the union boxes is 1:1. In experiment, we set both the height and width of the union boxes to 224 pixels. The fourth column are the heat maps for the selected pairs of objects by Grad-CAM.

In Figure 6, the relationships of examples I–IV are correctly predicted except the example V. From their heat maps, we can see that the features affecting visual manipulation relationships concentrate on the areas where the object pairs are in contact, which explains why our model can infer the manipulation relationships of objects. For the heat map of the example V, the features mainly concentrate on the screwdriver rather than the contact area of the two boxes. In other words, in the process of relational reasoning, we should pay attention to whether there is the obvious contact between the objects, and then the visual manipulation relationship is inferred according to the features of contact area. From examples I and III in Figure 6, we can see that the category of object relations has nothing to do with the characteristics of the object itself. In examples I and III, the banana is included in the object pairs, and the manipulation relationships are correctly predicted as “the banana is on the cup" and “the banana is on the notebook". However, the heat map is not concentrated on the banana. That is to say, even if the category of the object is not known, the visual manipulation relationship can be correctly predicted. This provides the possibility for the relationship prediction of unknown objects.

### 3.5. Robot Grasping Experiment

In order to verify the applicability of our method in the real environment, we carried out an experiment on the real robot. In experiment, a 6-DoF arm, AUBO-i5, is used as the experimental platform, and a depth camera, which is installed at the end of the arm, is used to collect RGB images.

**Figure 7:**
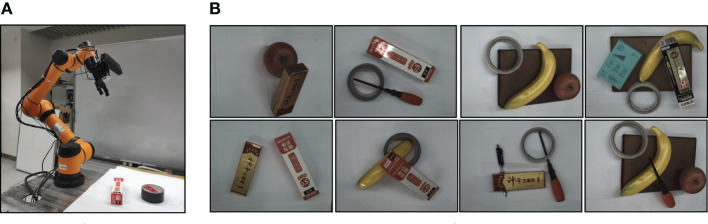
Robot grasping experiment. **(A)** Robot experimental environment, and **(B)** some examples for grasping manipulation.

To verify the generalization of the trained model, 100 images were collected and tested on our experimental platform. Each image contains two to five objects. Different models were tested on the platform. Figure 7 shows the complete experimental environment of AUBO-i5 and example images we collected. We use GVMRN and GVMRN-RF with ResNet10 and VGG16 to infer visual manipulation relationship. For the OR and OP metric, the reasoning accuracy can reach more than 82%. GVMRN-RF with VGG16 has the best performance that the OR and OP are 86.8 and 87.2%, respectively. For the IA metric, all of them are above 60%, and GVMRN-RF with VGG16 has the highest accuracy of 67%. Columns 6 to 9 in Table 3 show the IA performance of the images which contain different numbers of objects. When the number of objects is small, all the relationships in the image are easier to be inferred correctly. As the number of objects increases, the accuracy decreases, which is consistent with the results in Table 2.

**Table 3:** Results of visual manipulation relationship reasoning in robot experiment.

**Feature network**	**Model**	**OR(%)**	**OP(%)**	**Image-based accuracy (IA)**				
				**Total (%)**	**Object number per image**			
					**2**	**3**	**4**	**5**
ResNet101	GVMRN(ours)	82.4	83.6	60	18 / 22	19 / 31	14 / 26	9 / 21
	GVMRN-RF(ours)	84.3	84.5	62	19 / 22	19 / 31	**15** / 26	9 / 21
VGG16	GVMRN(ours)	85.5	85.9	63	19 / 22	20 / 31	14 / 26	10 / 21
	GVMRN-RF(ours)	**86.8**	**87.2**	**67**	**20** / 22	**21** / 31	**15** / 26	**11** / 21

*Each bold value represents the optimal result for each metric for different methods*.

The results show that our model can still achieve good relationship reasoning accuracy in real environments, which reflects the practicability and generalization capability of our model. But the overall test accuracy is slightly lower than that on the above experiment. The reason is that the objects we used are different from those in the dataset used in the above experiment, and the light conditions of image data are also different, because they were collected in different environments.

**Figure 8:**
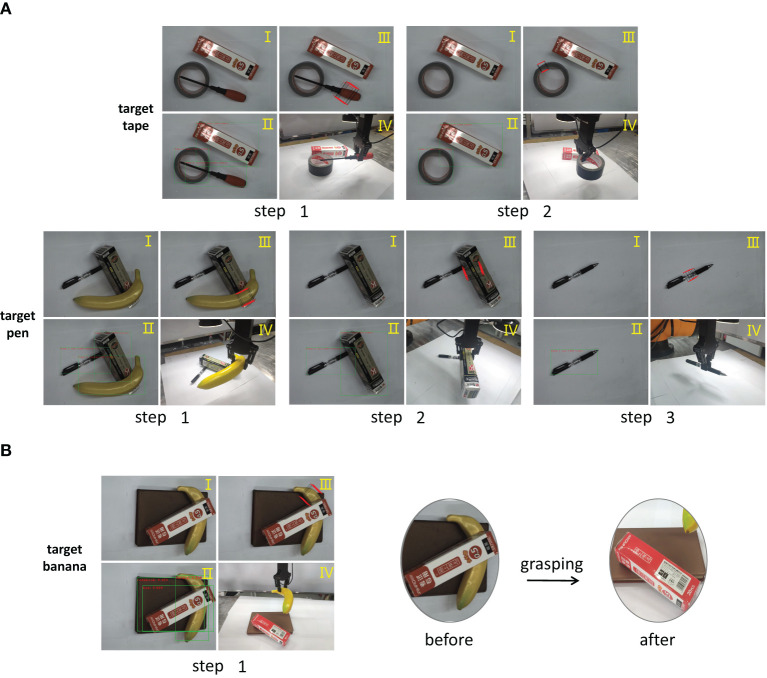
The examples of robot grasping experiment. **(A)** Grasping with GVMRN, **(B)** Grasping directly. Each grasping step includes (I) object detection, (II) manipulation relation reasoning, (III) grasping detection, and (IV) grasping the object with the robot arm.

To actually apply our method to complete the robot grasping process, the robot needs to identify the objects and inferring their manipulation order of objects. Moreover, the robot also needs to obtain accurate grasping position. In our experiment, the manipulator arm is vertically downward to grasp the object from a single angle, so we represent the grasping position as (*x, y, w, h*, θ), where (*x, y*) is the coordinate of the center point, (*w, h*) is the width and height of the grasp rectangle, and θ is the rotation angle with respect to the horizontal axis. We use the Multi-grab Detection model (Chu et al., 2018) to train the images with grasp annotation in the VMRD dataset (Zhang et al., 2020), and then directly use the trained model to obtain grasp candidates in the real environment. In the experiment, we add the depth information to calculate the position of the grab point in 3D space. Figure 8 shows the complete robot grasping process for different target objects in stacked scenes. The main difference between (A) and (B) is whether or not the robot adopts the reasoning mechanism.

As shown in Figure 8A, the robot detects the object and infers all the manipulation relationships and orders. For the tape example, the original image and the results of the detection and inference are shown, respectively in I and II of step one. We can see that if the tape is to be obtained, the screwdriver needs to be removed first. So the robot first makes grasp detection for the screwdriver and then performs the grasping manipulation. The results of both grasp detection for the screwdriver and robot execution can be seen from III and IV of step one. In step two, the robot performs the similar manipulation. We can see that the result of the manipulation order shows that the tape can be directly grabbed, and the robot successfully grasps the target object. For the pen example, we can see that the robot performs three grasp actions before it gets the target object. It can be concluded that the robot repeats four steps—object detection, manipulation order reasoning, grasp detection, grasp execution—until it grabs the target object. In the whole process, the grasping order inferred by the visual manipulation relationship guides the robot to obtain the target object efficiently.

However, for the banana example of Figure 8B, we can see that it is under the box and the robot grasps the banana directly after detecting it. In the right part of Figure 8B, the box is totally overturned due to the grasping actions. It can be imagined that if the box is fragile, it will be destroyed. If the orderly grasping mode is not used, the grasp candidate may be not optimal in the case that the target object is blocked, which leads to a failed grasping. Therefore, it is very important that orderly grasping is taken into account in real scenes. The above experiment shows that our method can guide the robot to grasp efficiently.

## 4. Conclusion

In this paper, we propose a graph-based visual manipulation relationship reasoning network for predicting manipulation order from RGB images. Our model focuses on collecting contextual information by graph convolutational network to output object relationships and manipulation order in object-stacking scenes. Besides, the relationship filtering network is built to prune out object pairs that are uncorrelated. Comparison experiments of our model with other state-of-the-art methods verify the effectiveness of our method. The verification experiment on the physical robot further shows the generalization and practicability of our proposed model. In future work, we will investigate how to achieve higher accuracy of relation reasoning, even if the number of the stacked objects increases. We also will further associate our method with other feature extraction models to improve real-time grasping performance.

## Data Availability Statement

Publicly available datasets were analyzed in this study. This data can be found at: http://gr.xjtu.edu.cn/web/zeuslan/visual-manipulation-relationship-dataset.

## Author Contributions

Conceptualization and manuscript writing were done by GZ. Experimental design and data analysis were done by JT. Manuscript writing and revision were done by HL. Review and commentary were done by WC and JL. All authors read and approved the final manuscript.

## Conflict of Interest

The authors declare that the research was conducted in the absence of any commercial or financial relationships that could be construed as a potential conflict of interest.

## Publisher's Note

All claims expressed in this article are solely those of the authors and do not necessarily represent those of their affiliated organizations, or those of the publisher, the editors and the reviewers. Any product that may be evaluated in this article, or claim that may be made by its manufacturer, is not guaranteed or endorsed by the publisher.
